# Molecular mechanisms of anthracycline induced cardiotoxicity: Zebrafish come into play

**DOI:** 10.3389/fcvm.2023.1080299

**Published:** 2023-03-10

**Authors:** Maryam Moossavi, Xiaoguang Lu, Joerg Herrmann, Xiaolei Xu

**Affiliations:** ^1^ Department of Biochemistry and Molecular Biology, Mayo Clinic, Rochester, MN, United States; ^2^ Department of Cardiovascular Medicine, Mayo Clinic, Rochester, MN, United States; ^3^ School of Traditional Chinese Medicine, Beijing University of Chinese Medicine, Beijing, China

**Keywords:** zebrafish, anthracyclines, cardiotoxicity, mitochondria, apoptosis

## Abstract

Anthracyclines are among the most potent chemotherapeutics; however, cardiotoxicity significantly restricts their use. Indeed, anthracycline-induced cardiotoxicity (AIC) fares among the worst types of cardiomyopathy, and may only slowly and partially respond to standard heart failure therapies including β-blockers and ACE inhibitors. No therapy specifically designed to treat anthracycline cardiomyopathy at present, and neither is it known if any such strategy could be developed. To address this gap and to elucidate the molecular basis of AIC with a therapeutic goal in mind, zebrafish has been introduced as an *in vivo* vertebrate model about a decade ago. Here, we first review our current understanding of the basic molecular and biochemical mechanisms of AIC, and then the contribution of zebrafish to the AIC field. We summarize the generation of embryonic zebrafish AIC models (eAIC) and their use for chemical screening and assessment of genetic modifiers, and then the generation of adult zebrafish AIC models (aAIC) and their use for discovering genetic modifiers *via* forward mutagenesis screening, deciphering spatial-temporal-specific mechanisms of modifier genes, and prioritizing therapeutic compounds *via* chemical genetic tools. Several therapeutic target genes and related therapies have emerged, including a retinoic acid (RA)-based therapy for the early phase of AIC and an autophagy-based therapy that, for the first time, is able to reverse cardiac dysfunction in the late phase of AIC. We conclude that zebrafish is becoming an important *in vivo* model that would accelerate both mechanistic studies and therapeutic development of AIC.

## 1. Introduction

Since the 1990s there has been a steady decline in cancer-related mortality, and as a consequence a steady increase in the number of cancer survivors (1). With this development has come a deeper understanding of the significance of the side effects of cancer therapy, some of the most impactful being cardiovascular in origin (2). While the number and types of cancer therapies has been expanding remarkably over the years, anthracyclines remain the class of chemotherapeutics most tightly linked to long-term cardiotoxicity (3–11). Much of the focus on the topic of anthracycline-induced cardiotoxicity (AIC) has been on prevention before and during chemotherapy. Efforts to improve anthracycline cardiomyopathy once it has developed are limited.

Zebrafish is emerging as a vertebrate model with ample potential to address these gaps. This model allows for high-throughput genetic screening and thereby critical insight into molecular pathways and therapeutic strategies to rectify defects and recover biological function. In the current review, we will first introduce our current understanding of key underlying mechanisms of AIC. We will then summarize recent studies in zebrafish that have led to advances in our understanding of AIC and point to novel therapeutic strategies.

## 2. Anthracyclines structure, history and clinical use

Anthracyclines are a family of natural antibiotics with planar four-ringed structures comprising a quinone and an amino sugar group. As part of a comprehensive search for anticancer drugs, the archetypal compounds of this class were extracted from a mutated strain of the soil bacterium Actinobacteria Streptomyces Peucetius (12). Daunorubicin (daunomycin) was first discovered in 1964 (13, 14), followed by doxorubicin (Adriamycin) in 1969 (15). They were clinically tested soon after, and by the early 1970s, they had been registered. Since then, they have been marketed and become the anthracyclines' prototypes (16). Various anthracycline derivatives have been created and studied over the last five decades, comprising epirubicin (doxorubicin's semisynthetic epimer), idarubicin (daunorubicin's chemical analog), mitoxantrone (doxorubicin's stereoisomer) and valrubicin (doxorubicin's semisynthetic analog) (7). These are the foundation for several chemotherapy plans in breast cancer, lymphoma, leukemia, bladder and sarcomas (9).

## 3. Molecular mechanisms of AIC

Anthracyclines lead to cardiomyocyte death, progressive patchy myocardial necrosis, and ultimately multifocal myocardial fibrosis. This sequence is linked to the progression across the heart failure stages from exposure to asymptomatic cardiomyopathy, clinical heart failure and eventually end-stage heart disease (11). The molecular mechanisms that underlie AIC are complex and involve multiple pathological processes as reviewed in the following.

### 3.1. Anthracyclines transportation in cardiac cell

The strategy for anthracycline neutralization and detoxification in a cardiac cell is diverse, and failure of involved proteins can result in anthracycline accumulation. The solute carrier (SLC) 28A3 transporters (also known as sodium-coupled nucleoside transporters) are expressed in the human heart and are used by anthracyclines as an entry point (17). The cardioprotective effects of *SLC28A3* has been recapitulated in human induced pluripotent stem cell model, and desipramine, a SLC competitive inhibitor, has been identified to exert protective effects on AIC (18). Moreover, ATP-binding cassette (ABC) proteins are involved in exporting multiple chemotherapeutics, such as anthracyclines, from cardiac cells using energy derived from ATP hydrolysis. ABCC1-ABCC2-ABCC5, as prominent proteins of this type, are membrane bound transporters highly expressed in human cardiac cell (9, 19). In addition, the following genes listed in Table 1 are among the most prominently involved.

**Table 1:** Prominent genes involved in anthracyclines transportation.

Gene name	Protein Function
Solute carrier 28A3 transporters (SLC28A3)	• Anthracyclines entry point (17).
ATP-binding cassette proteins (ABCC1-ABCC2-ABCC5)	• Exporting anthracyclines using ATP hydrolysis (9, 19)
Carbonyl reductases (CBR)	• Catalyzing anthracyclines into toxic C-13 alcohol metabolites during anthracycline metabolism (20, 21)
Uridine Diphosphate glucuronosyltransferase family one member A6 (UGT1A6)	• Detoxification glucuronidation pathway. • Recruits and converts poisonous anthracycline metabolites into water-soluble and excretable compounds (21).
Sulfotransferase family cytosolic member 2B1 (SULT2B1)	• Conjugates sulphate to anthracycline medicines, enhancing their water solubility and facilitating excretion (22).
Glutathione S-transferase M1 (GSTM1)	• Catalyzing the detoxification of many carcinogens drugs including anthracyclines (23).

### 3.2. Nucleus effects of anthracyclines

The proteasome is thought to be involved in transporting anthracyclines from the sarcoplasm to the nucleus (24). Anthracyclines through the planar ring system intercalate the DNA bases and the amino sugar region, which prevents DNA replication and transcription. Inhibition of Topoisomerase-2 (Top2alpaha in cancer cells) interferes with the release of DNA supercoils and the ternary complex of anthracycline, topoisomerase and DNA inhibits the re-ligation of double-stranded breaks leading to their persistence. Further, doxorubicin-induced oxidative stress facilitates phosphorylation and activation of ERK and p53 (25). Subsequently, activated p53 enters the nucleus and up-regulates the expression of Bax and PUMAα. PUMAα can not only directly promote the translocation of Bax to mitochondria, but also indirectly activates Bax by competitive binding of Bcl-xL protein (26). Also, the activated p53 protein facilitated down-regulation of transcription factor GATA-4, which results in the lower expression of the Bcl-2 protein (27). The above-mentioned factors cause Bax to form pores in the mitochondrial outer membrane, leading to release of cytochrome C (Cyt c), which stimulates the activation of caspase-9/-3 and thereby cell apoptosis (28–30).

### 3.3. Mitochondrial effects of anthracyclines

There is considerable overlap between the mechanisms accounting for anticancer effects and cardiotoxicity though distinct differences exist. Anthracyclines, for example, block topoisomerase-2 beta in cardiomyocytes and alpha in cancer cells and have a preference for mitochondria in cardiomyocytes. Mitochondrial injury appears to be a key component of anthracycline-related cardiotoxicity, and has been hypothesized as the cause of the long-term risk of cardiomyopathy linked with anthracycline exposure (2). According to studies, the expression of the transferrin receptor (TfR) is up to five times higher in cancerous tissue than in healthy tissue (31), DOX-Tf compound should be able to increase the intracellular concentration of medicines in breast cancer cells, assisting in the treatment of chemoresistance (32). As previously reported, DOX conjugation with Tf markedly increased cytotoxicity in human leukemia cells, both DOX-sensitive and DOX-resistant, as well as those derived from solid tumor cells (33). According to earlier studies, Tf-bound DOX triggers the TRAIL-dependent, extrinsic apoptosis pathway, which results in programmed cell death. TNF- and other cytokines are present, demonstrating a connection between the conjugate's pro-inflammatory effects and its cytotoxicity (34, 35).

#### 3.3.1. Role of cardiolipin

Cardiolipin (CL) is a major phospholipid located in the inner membrane of the heart mitochondria and has a high structural affinity for anthracyclines. The medicine can diffuse passively *via* cell membranes and disseminate into mitochondria due to its lipophilic substances. Afterward, anthracyclines can attach to the cardiolipin, and be reduced from its quinone form to a semiquinone by NADH dependent enzymes. The semiquinone changes to a quinone state by donating an electron to an oxygen (O_2_) molecule, creating a superoxide anion $\left(O_{2}^{-}\right)$. The mitochondrial electron transport chain (ETC) is destroyed after repeated redox cycles, and many reactive oxygen species (ROS) are created. This damage cycle causes externalization of CLs from the inner membrane to the outer membrane *via* nucleoside diphosphate kinase (NDPK-D). Comprehensive studies have declared that the chief corner-stone event in initiation of the pro-apoptotic event is CL oxidation catalyzed by the peroxidase function of an intermembrane space hemoprotein, Cyt c. CL-ox interacts with essential players of autophagy, Beclin1, and recruits the autophagic apparatus by its interaction with LC3. On the other hand, apoptotic signaling pathways rely on CL-ox as a binding scaffold for apoptotic components including tBid, Bax, and caspase-8 to be recruited. CL-ox has an essential role in the processing of procaspase-8 to the active form. Caspase-8 activation leads to BH3 interacting domain death agonist (BID) cleavage, carboxynl fragment (tBID) activation and translocation into mitochondria for oligomerization of Bax and Bak. Then, cytochrome c is detached from CL-ox and released into the cytosol space and activates caspase 9 to initiate apoptosis (36, 37).

#### 3.3.2. Role of free radical attack

Cardiomyocytes are extremely sensitive to ROS-induced injury compared to other cell types, which can be described by the heart's high demand for the metabolism of oxidative substrates to pump energy through a large number of mitochondrial (11). Anthracyclines generate ROSs by two enzymatic and non-enzymatic pathways:

(i) Enzymatic pathways
  • Comprising single-electron reduction of the quinone ring and converting into semiquinone under the action of nicotinamide adenine dinucleotide phosphate (NADPH) oxidase (NOXs) is highly expressed in the cardiomyocyte mitochondrial and, has a key role in the anthracycline-induced production of ROS in the myocardium which can alter the risk of AIC. In line with this hypothesis, NADPH oxidase knockout mice revealed decreased risk of AIC in a mouse model (38). Studies showed that the activity of NOXs in cardiomyocytes treated with doxorubicin was augmented and the expression of NOX2/NOX4 mRNA was unregulated, suggesting NOXs activation might be a vital mechanism for cardiomyocyte apoptosis and injury caused by anthracycline (38).
(ii) Non-enzymatic pathways
  • The iron in the mitochondria is involved in forming ROSs. The Fenton reaction can convert hydrogen peroxide generated by anthracycline metabolism into a hydroxyl radical with the availability of free iron (iron-catalyzed Haber-Weiss reaction). Alternatively, doxorubicin's positive charge facilitates a strong affinity for iron, bringing about the formation of the Dox-Fe complex, which disrupts iron metabolism. Physiologically, Nrf2, a redox-sensitive transcription factor, creates a complex in the cytoplasm with the negative regulator Kelch-like epichlorohydrin-associated protein-1 (Keap1) and impeding its function (39). Anthracycline-induced ROS uncoupled Nrf2-Keap1 complex and facilitates it nucleus translocation to link with an antioxidant response element (ARE) in the nucleus and causing hemoxygenase-I up-regulation. Therefore, heme is degraded in the heart as a result of this overexpression and huge amount of iron release and deposit in the mitochondria. This phenomenon starts the process of cardiomyocyte ferroptosis (25). On the other hand, the oxygen free radicals caused by doxorubicin can inhibit PI3K expression/Akt phosphorylation and thereby control microfilament rearrangement, which affects the depolymerization of actin and interferes with the nuclear translocation of Nrf2 (40, 41). HO-1 expression reduces which exacerbates oxidative stress condition an encouraging cardiomyocyte apoptosis (41, 42). The free iron content levels in normal physiology are not sufficient to bind DOX, and thus cardiotoxicity does not occur. Conversely, the only licensed cardio-protective medication against anthracycline cardiotoxicity is Dexrazoxane (DXZ), an iron chelator.
  • Free radicals induced by anthracycline disrupt mitochondrial calcium homeostasis. Decreased mitochondrial calcium load capacity due to ROS can lead to high concentrations of Ca^2+^ in the cytoplasm, activating calcineurin, which inhibits PI3K/Akt/mTOR pathway activation and promotes dephosphorylation, activation and nuclear translocation of nuclear factor of activated T cells (NFAT). In cardiomyocytes, NFAT interacts with the promoter region of the FasL gene in the nucleus, and up-regulates transcription of FasL mRNA resulting in increased production of soluble and membrane FasL proteins. Fas triggers apoptosis by activating caspase-3 by binding to Fas-associated protein with death domain (FADD) and caspase-8 (42).

### 3.4. Sarcoplasmic reticulum effect of anthracyclines

Homeostasis in the sarcoplasmic reticulum can break down due to various physical and chemical factors. Anthracyclines create ROS by single electron transfer under the action of SR flavoenzymes such as NADPH-cytochrome P450 reductase, which can disrupt Ca^2+^ homeostasis in the SR of cardiomyocytes and impair the function of Ca^2+^-dependent chaperone proteins and enzymes; hence, triggering the unfolded protein response (UPR). In fact, anthracycline-induced ROS and RNS can suppress the expression of sarco/endoplasmic reticulum Ca^2+^-ATPase2 (SERCA) resulting in decreased Ca^2+^ absorption across a concentration gradient from sarcoplasm to SR. As a result, there is an increase in the phosphorylation level of Ca2+/calmodulin-dependent protein kinase II (CaMK II), an essential kinase that regulates Ca^2+^ homeostasis, and over-activated CaMK II abnormally up-regulates the phosphorylation of SR calcium channels and calcium pumps, leading to calcium imbalance (43). Therefore, excessive accumulation of abnormal proteins due to Ca2+-dependent chaperone protein impairment, activates SR transmembrane sensors including protein kinase-like endoplasmic reticulum kinase (PERK), transcription factor 6α (ATF6α) and inositol-requiring kinase 1α (IRE1α). Thereafter, luminal SR chaperone, glucose-regulated protein 78 (GRP78; known as immunoglobulin-binding protein or BiP) is activated and released from these sensors and allows dimerization and auto-phosphorylation of PERK as well as IRE1α, and translocation of ATF6α and further processing in the nucleus. PERK is a kinase enzyme which phosphorylates and inactivates the elongation initiation factor (eIF2a), leading to a broad reduction in protein translation, which in turn leads to the translation activating transcription factor 4 (ATF4) and transcription factor C/EBP homologous protein (CHOP) (44, 45). The abnormal expression of CHOP can change Bax/Bak by inhibiting Bcl-2, leading to increased mitochondrial outer membrane permeability, which makes large amounts of Ca^2+^ leak from the SR lumen into sarcoplasm. Subsequently, calmodulin is activated and then mediates the activation of caspase-12, which further activates caspase-9/-3 and finally initiates apoptotic pathway in cardiomyocyte (44).

## 4. Contribution of zebrafish embryos to AIC

Danio rerio, a bony fish (teleost), resident of freshwater rivers “Ganges mainly” of Himalayan area of South Asia has been recruited for biological studies. These fish belong to the Cyprinidae family and class of Actinopterygii (ray-finned fishes) sharing an outstanding genetic and physiologic similarity with humans (46). The genome of zebrafish, shows almost 70% homology with human, and over 80% of the disease-related genes. Zebrafish produce approximately 200 fertilized eggs per mating. Their extrauterine growth is rapid—the major organs of the zebrafish are developed by 5 day-post-fertilization (dpf). They are tiny (1–1.5 inch) and require a low-cost diet (47). The optical clarity of embryo and larvae enables time-lapse non-invasive fluorescent imaging and protein/cell marker tracking. Together with its powerful forward genetic, reverse genetic and chemical genetic tools, zebrafish have been leveraged for deciphering molecular mechanisms of AIC (48, 49).

### 4.1. Embryonic zebrafish models for AIC (eAIC)

Cardiotoxicity in zebrafish embryos can be noted when doxorubicin (DOX) is administered at different time windows ranging from hatching (0–3 dpf) (50) to post-hatching larva (after 3 dpf) (51). To avoid early cardiogenesis, Liu et al. (2014) incubated zebrafish embryos with solutions containing 100 µM doxorubicin at 1 day post-fertilization (dpf), when the heart had formed and circulation had begun (50). Given that zebrafish embryos are encircled within a protective chorion before 3 dpf, and most drugs are enriched inside the chorion (52), some researchers prefer to use either post-hatch larvae that naturally remove the chorion or dechorionated embryos for modeling AIC. An alternative method to incubation is to deliver anthracyclines *via* intrapericardial injections (53). Consistent cardiac phenotypes have been noticed among different delivery methods, including pericardial edema, impaired cardiac contractility, decreased blood ﬂow, and increased apoptosis (50). We termed these models as embryonic AIC models (eAIC) in this review. Besides doxorubicin (DOX), Han et al. assessed cardiotoxicity incurred by other anthracyclines in zebrafish embryos, including daunorubicin (DAU), pirarubicin (PIRA), and epirubicin (EPI), as well as a liposome encapsulated DOX. They found that cardiotoxicity of DOX can be partially attenuated by the liposome packaging, while DAU caused the least cardiotoxicity. While DOX reduces heart rates significantly, the other four anthracyclines do not (54).

### 4.2. Utilization of eAIC to discover therapeutic strategies

An attractive attribute of the eAIC models is their powerful chemical genetic capacity, which enables rapid assessment and screening of large number of compounds. eAIC models have been used widely to assess candidate therapeutic compounds that have been reported to exert therapeutic effects on other types of cardiotoxicity and/or cardiomyopathy and to elucidate the underlying mechanisms. D006-induced cardioprotection on eAIC was noted, which may be facilitated by maintenance of mitochondrial biogenesis and augmentation of HO-1 expression (55). Derivative of danshensu and tetramethylpyrazine–DT-010 might rescued cardiomyocytes in eAIC by decreasing ROS generation and preventing cell death, which may be facilitated through the stimulation of the PGC-1α/Nrf2/HO-1 pathway (56). Cardioprotective functions of synthesized Isosteviol analogues on eAIC could be exerted through blocking NF-κB signaling pathway and rising the mitochondrial action (57). Cardioprotective effects of allium extracts, including allium flavum and allium carinatum, have been noted (58). Tanshinone IIA, a component of *Salvia miltiorrhiza*, exerts cardioprotective effects on eAIC by increasing the autophagic flux *via* autophagosome formation and autolysosome clearance through the Beclin1/LAMP1 molecular pathway (59). A009, a polyphenol-rich Olive mill wastewater (OMWW) extract, was able to counteract the doxorubicin-induced cardiotoxic effects in the zebrafish eAIC model, which is consistent to its cardioprotective activities on rat and human cardiomyocytes. Intriguingly, this waste material from extra-virgin olive oil (EVOO) processing is able to increase the effects of breast cancer chemotherapy *via* its antiangiogenic and antiproliferative activities (60). While all of these aforementioned compounds are able to rescue cardiac dysfunction to a certain degree, therapeutic target genes for these compounds remain elusive and further mechanistic studies are needed to translate these discoveries to AIC patients. In addition to chemical genetic studies, eAIC has been used as an animal model to assess gene modifying effects. Yamashita et al. used CRISPR–cas 9 technique to knock down the key antioxidant responsive gene–*nuclear factor erythroid 2-related factor 2a (nrf2a).* They found that Nrf2a-deficient zebrafish showed increased sensitivity to DOX-induced oxidative stress, prompting further studies of this gene as a beneficial modifier to AIC (61).

Besides testing known candidate therapeutic compounds and genes, eAIC models have been successfully utilized to discover new therapeutic antidotes. Dr. Peterson's laboratory completed two non-biased screens of compound libraries. Using an eAIC model whereby 1 dpf zebrafish embryos was treated with DOX for 48 h, they assessed 3,000 small molecules from the Prestwick and Spectrum chemical libraries and identified visnagin (VIS) and diphenylurea (DPU) as two hits. Through studying effects of mitochondrial malate dehydrogenase 2 (MDH2) inhibitors on the zebrafish eAIC model, the authors propose that visnagin's cardioprotective effect is associated with MDH2 modulation (50). Further studies uncovered cytochrome P450 family 1 (CYP1) as an important genetic factor for AIC (62). This conclusion was later confirmed by screening 2,271 small molecules from a proprietary, target-annotated tool compound collection. A total of 120 compounds with anti-cardiotoxicity effects were identified with seven of them being extremely efficacious (63). Interestingly, all seven highly effective compounds exhibited inhibitory activity towards cytochrome P450 family 1 (CYP1), despite their structural differences. In addition to the cardioprotective effects of CYP1 inhibitors, a zebrafish CYP1A mutant was found to be resistant to DOX cardiotoxicity, providing genetic evidence to support CYP1 as a therapeutic target gene for eAIC (63).

## 5. Contribution of adult zebrafish to AIC

### 5.1. Adult zebrafish models for AIC (aAIC)

Despite a convenient *in vivo* model with high throughput, eAIC cannot fully recapitulate the progressive pathological process that often occurs months or years post chemotherapy in human patients. To address this shortcoming, Ding and co-workers reported the first DOX-induced cardiotoxicity model in an adult zebrafish (64). They injected a single bolus of DOX intraperitoneally into zebrafish of two months to one-year of age. Significant mortality was noted 8–12 weeks later, when their hearts showed cardiomyopathy hallmarks including activation of fetal gene expression, muscular disarray, and myofibril loss (64). Ma et al. presented a detailed summary of their injection protocol, and described two intraperitoneal injection methods, aiming to reduce the variation that might be associated with internal organ damage (65). Later, Wang et al. introduced a third intraperitoneal injection method, whereby the needle penetrated the ventral midline between the pectoral fins (66). Using this new method, significant mortality is avoided, while the fish still manifests severe cardiac dysfunction at 8 weeks post injection.

Because aAIC was among the first cardiomyopathy models in adult zebrafish, this model has been used extensively to develop phenotyping tools. When the aAIC model was first reported in 2011, high-frequency echocardiography (HFE) was not available and survival rate at 8 week post injection (wpi) or later has been used as a noninvasive index to assess aAIC. The other non-invasive index was critical swimming speed (Ucrit) that was measured using a swimming tunnel, which corresponds to exercise capacity measured by treadmill testing in rodent models or patients (67, 68). Later, Zhang et al. developed a Langendorff-like ex vivo system to measure cardiac function in an isolated adult zebrafish heart, which was used to quantify ejection fraction (EF) in aAIC models (69). A transient decline of EF was noted during the first week post-injection (wpi), reflecting early stage acute cardiac damage. EF then recovered after 1 wpi, only to drop again after 4 wpi, with a statistically significant decease at 8 wpi, reflecting a later cardiomyopathy phase of the aAIC model. Packard et al. developed an automated segmentation approach based on histogram analysis of raw axial images acquired by light-sheet fluorescent imaging (LSFI) to establish rapid reconstruction of the 3-D zebrafish cardiac architecture (70). They noted reduction of heart size in the early phase of the aAIC model, which is consistent with a recent report in human AIC patients (71, 72). Later, the same laboratory developed a semiautomated, open-source method—displacement analysis of myocardial mechanical deformation (DIAMOND)—for quantitative assessment of 4D segmental cardiac function. They demonstrated that basal ventricular segments adjacent to the atrioventricular canal display the highest 3D displacement and are also the most susceptible to doxorubicin-induced injury (73). The introduction of high frequency echocardiography enables reliable non-invasive quantification of ejection fraction in an adult zebrafish heart (74). The technology has been applied to the aAIC model, which was able to non-invasively report reduced EF at the late phase of aAIC (66).

### 5.2. Comparison between zebrafish and rodent aAIC models

One of the most clinically relevant aAIC models in mice is created by administering low dosage doxorubicin (5 mg/kg) intravenously (IV) four times per week (75, 76). This dosage was chosen based on the pharmacokinetics of DOX in mice following a single dose of 5 mg/kg, which is equivalent to the pharmacokinetics of DOX in cancer patients after a normal dose of DOX therapy (60 mg/m^2^) (75). In mice, the IV route was preferred to the intraperitoneal (IP) route because IP injection of DOX causes damage, fibrosis, and consequent malaise, anorexia, weight loss, and noncardiac decease (75, 77–79). Because 20 mg/kg doxorubicin was administered in the zebrafish aAIC model ages 2 months up to 1 year, this dose is the same with the accumulative dose in the mouse aAIC model (64). In the zebrafish aAIC model, reduced ejection fraction can be first noted at 4 weeks post injection (wpi) and the end functional readout is typically carried at 8 wpi, which is similar to the mouse aAIC model, whereby reduced ejection fraction is first noted at 2 wpi and the end functional readout is typically done at 7 wpi when cardiac function has become stable. Because adult zebrafish have strong regenerating abilities in their heart muscle, which mammals lack (80), it was unclear if the zebrafish aAIC model has distinct responses to DOX at the cellular level. To address this concern, Ding et al. revealed that while cardiomyocyte hypertrophy contributes to cardiac remodeling, cardiomyocyte hyperplasia does not, as evidenced by unaltered PCNA index and 5-bromo-2-deoxyuridine (BrdU) incorporation (64). Based on these data, they concluded that the pathogenesis in the zebrafish aAIC model is likely conserved with that in mouse aAIC, without incurring significant compensational cardiac regeneration.

### 5.3. A forward genetic screening strategy in aAIC enables systematic discovery of AIC susceptibility genes

The generation of aAIC models and the development of phenotyping tools in zebrafish paved the way towards leveraging powerful zebrafish genetic tools to study AIC. A non-biased forward genetic approach has been recently established, which enables the discovery of modifying genes for AIC, i.e., genes that either accelerate or slow down the progression of AIC (82, 83). The screen was built on a gene breaking transposon (GBT)-based insertional mutagenesis system that is proven to effectively disrupt tagged genes (84, 85). Besides efficient gene disruption in each GBT mutant line, the expression pattern of the endogenous gene can be reported by a RFP fluorescent protein reporter. Around 1,000 GBT mutant lines have been generated at Mayo Clinic, ∼20% of which manifest visually noticeable cardiac expression, resulting in the generation of a zebrafish insertional cardiac (ZIC) mutant collection (86). Ding et al. stressed heterozygous ZIC lines with DOX to search for genetic factors that modify the survival and/or cardiac function of aZIC (86). By screening 609 GBT lines, they identified 4 candidate aAIC-modifying genes, including *GBT0002/sorbs2b, GBT0136/ano5a, GBT0411/dnajb6b (L)* and *GBT0419/rxraa*.

To query where the screen is effective in discovering meaningful genes in AIC and/or cardiomyopathy, follow up studies of these 4 GBT mutant lines and affected genes were carried out. (1) *ANO5* is a known dilated cardiomyopathy (DCM) causative gene (87) (2) Studies of a mouse *Sorb2* knock out (KO) mutant confirmed the modifying effects of heterozygous *Sorb2* KO on AIC (88). Interestingly, *Sorbs2* encodes an intercalated disc (ICD) protein and homozygous *Sorb2* KO mutant manifests arrhythmogenic right ventricular cardiomyopathy (ARVC)-like phenotypes, including enlarged right ventricle and cardiac arrhythmia. Preliminary human genetic studies identified likely pathogenic variants from two ARVC patients, supporting *SORBS2* as a candidate ARVC gene (88). (3) The *DNAJB6* gene encodes a member of the J protein family, which serves as a chaperone molecule to aid folding and quality control of the protein (89). Alternate splicing of the same gene results in two DNAJB6 isoforms: a short DNAJB6(S) isoform that is encoded by the first 6 exons, and a longer DNAJB6 (L) isoform that adds two additional exons to the 3′ end of the gene (86). Different from the short *DNAJB6(S)* isoform that is a causative gene for limb girdle muscular dystrophy (LGMD) (90, 91), *DNAJB6(L)* is a cardiac-enriched isoform (86). Pathogenic sequence variants were found in dilated cardiomyopathy (DCM) patients, and a transgenic fish line harboring the human variant DNAJB6 (L)-S316W exerts deleterious modifying effects on aAIC in zebrafish. In contrast to deleterious modifying effects of *GBT411/dnajb6(L), Tg(DNAJB6(L))* exerts therapeutic effects on aAIC (86). The therapeutic benefits of *Dnajb6 (L)* overexpression were confirmed in a mouse AIC model by using a AAV9-based gene delivery system. (4) In contrast to the other 3 GBT lines that exert deleterious modifying effects, *GBT419* exerts salutary modifying effects on aAIC. Detailed molecular studies unveiled that the salutary modifying effects of *GBT0419* can be ascribed to gain-of-function of *retinoid x receptor alpha a (rxraa)* in endothelial cells, but not in cardiomyocytes or in epicardial cells (67). The statement that *RXRA* is a new susceptibility gene for AIC was supported by a GWAS analysis of 1,191 patients with early-stage breast cancer treated with DOX (67).

**Figure 1:**
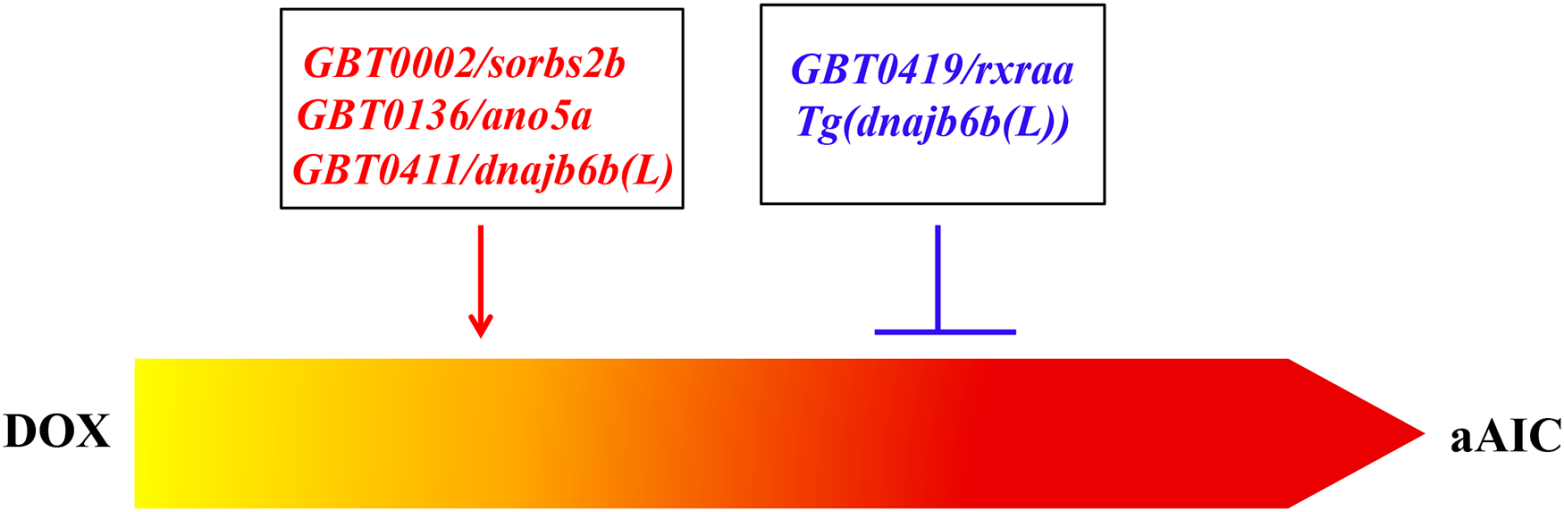
Both deleterious modifiers and salutary modifiers of AIC can be identified by using a forward mutagenesis screening approach. While *GBT0002/sorbs2b, GBT0136/ano5a,* and *GBT0411/dnajb6b(**L**)* were identified as deleterious modifiers, *GBT0419/rxraa* was identified as a salutary modifier. Mechanistic studies of *GBT0411/dnajb6b(**L**)* identified *Tg(dnajb6b(**L**))* that exerts salutary modifying effects on AIC (86).

In summary, follow-up studies of 4 GBT lines from a pilot screen confirmed important roles of these genes in AIC. In addition to modifying effects on AIC, many genes turned out to be susceptibility genes in cardiomyopathies, and some genes could be potential therapeutic target genes (Figure 1). This series of work established a new method for discovering genetic factors for AIC, which differs from the ongoing technologies such as genome-wide association studies (GWASs) in humans and quantitative trait locus analyses in rodents. There are significant limitations with these statistics-based methods, which often point to sizable genomic regions that cover a large number of candidate genes, making it difficult to confidently establish genotype-phenotype relationships (92). Conversely, the identity of each modifier gene identified from an aAIC-based forward genetic screen can be unambiguously determined. Therefore, the zebrafish aAIC model potentially opens the door to systematic discoveries of genetic modifiers of AIC, which could be achieved by scaling this forward genetic strategy to the whole genome.

### 5.4. A time-dependent mechanism has been suggested from studies of aAIC

Using the aAIC model, Ding et al. demonstrated that a heterozygous *mTOR* mutant exerts therapeutic benefits on aAIC, providing the first genetic evidence on cardioprotective effects of mTOR inhibition on AIC (64). During this study, dynamic changes in mTOR signaling were noted over the dynamic course of aAIC. Consistent with this notion, they found that rapamycin, a mTOR inhibitor, exerts deleterious effects in the early stage of aAIC, which is opposite to cardioprotective effects in the late stage (66). Similar to mTOR signaling, biphasic changes in autophagy activity were noted in the aAIC model: increase in the early and decrease in the late stage. In line with time-dependent signaling changes, overexpression of Atg7, a core autophagy protein, exerts deleterious effects in the early stage, but salutary effects in the late stage of aAIC. Next, Ding et al. leveraged to use the efficient zebrafish model for screening FDA-approved drugs with autophagy activating function (FAA). They reported that top-ranking FAAs are able to revert declined cardiac function in the late phase of aAIC, but exert deleterious effects in the early phase (66).

**Figure 2:**
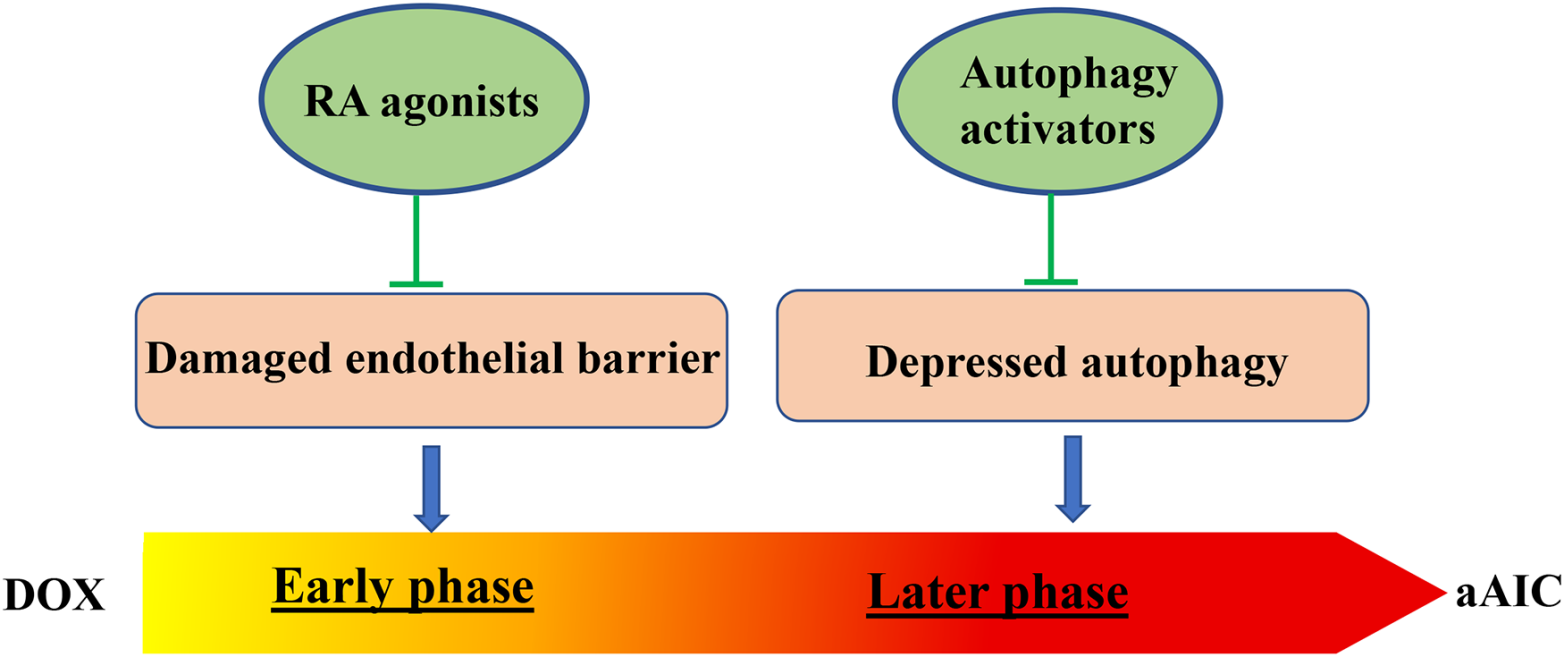
Two time-dependent therapeutic avenues have been suggested for aAIC. While RA agonist have been identified to exert therapeutic effects at the early phase of aAIC, autophagy activators have been identified to exert therapeutic effects at the later phase of aAIC (66, 67).

Besides autophagy, a time-dependent effect was also reported for RXRA-based therapy (67). The therapeutic effects of RXRA agonists only occur when administered during the early, but not the late, phase of AIC. Together, these studies in the *in vivo* zebrafish model raised an important concept that pathological signaling in the late phase of AIC can be quite different from, or even opposite to, the signaling in the early phase. Different types of therapeutic strategies must be designed to treat the early phase and late phase of AIC, respectively (Figure 2).

## 6. New therapeutic avenues for AIC have emerged from zebrafish studies

At present, AIC patients are mainly treated with universal heart failure therapies such as beta-blockers and angiotensin-converting enzyme inhibitors and as few as only 10% may experience complete recovery of their cardiac function (93). Dexrazoxane (DXZ) is the only approved AIC-specific agent but approved only for patients with metastatic breast cancer who benefit from additional anthracycline therapy after having already received 300 mg/m^2^ (94–96).

As such, there is no specific mechanism-based therapy to reverse chronic AIC. As outlined earlier, the introduction of zebrafish as an animal model of AIC has identified the following therapeutic target genes as potential therapeutic revenues for AIC:

1) *CYP1,* identified from compound screens using the eAIC model: CYP1 encodes a group of highly conserved monooxygenases responsible for the metabolism of environmental toxicants, including polycyclic aromatic hydrocarbons similar in structure to DOX. The lead compound is visnagin (62), and additional seven compounds have been identified (62, 63).
2) *RXRA,* discovered from a mutagenesis screen that identified *GBT419* as a salutary aAIC modifier: Overexpression of RXRA in endothelial cells is therapeutic in the early phase of aAIC. Isotretinoin and bexarotene, two FDA-approved RA activating compounds, have been identified as top compounds with therapeutic effects. Mechanistically, it is proposed that retinoic acid (RA) activation in endothelial cells repairs the damaged tight junction incurred by DOX that compromises the barrier function of endocardial cell layer to slow down the penetration of DOX from the blood to myocardium (67).
3) *DNAJb6 (L),* discovered from a mutagenesis screen that identified *GBT411* as a deleterious aAIC modifier: Overexpression of DNAJb6 (L) in cardiomyocytes exerts therapeutic effects on AIC in both zebrafish and mouse models (81). Mechanistically, it has been suggested that unfolded protein response (UPR) signaling is a critical downstream signaling. UPR inhibitors such as phenylbutyrate, a FDA approved drug, seems effective in treating aAIC (86).
4) *mTOR,* discovered from a candidate gene approach that was prompted by therapeutic effects of rapamycin (64): *mTOR* inhibition exerts therapeutic effects in the late but not the early phase of aAIC. Its therapeutic effects is likely owing to its functions in protein quality control (PQC) pathways such as autophagy.
5) *Atg7,* discovered during a follow up study of therapeutic effects of mTOR inhibition: Overexpression of ATG7 in cardiomyocytes exerts therapeutic effects in the late, but not the early phase of AIC. Pravastatin and spironolactone have been identified as top FDA-approved autophagy activators (FAAs) that might reverse cardiac dysfunction in aAIC *via* restoring the suppressed autophagic flux (66).

## 7. Conclusion and perspective for future research efforts

Since the introduction of zebrafish AIC models, the last decade has witnessed significant contributions of this animal model to genetic underpinning and therapeutic development of AIC. The possibility of conducting non-biased genetic and compound screens in this efficient vertebrate model creates unprecedented opportunity to discover novel therapeutic target genes and to develop mechanism-based therapeutic strategies. Spatial-temporal specific mechanisms can be deciphered by using genetic tools in this simple *in vivo* animal model, prompting zebrafish an efficient *in vivo* model to assess discoveries made in *in vitro* cell culture models. Because signaling pathways can be quite different in early vs. late phase of AIC, studies in an *in vivo* animal model is critically important, which would resolve many conflicting results in this field. Based on the rapid progress in the last decade, it is predicted that zebrafish will continue to advance the AIC field at the following three fronts:

1) The unique mutagenesis screening approach in zebrafish will continue to yield new genetic factors of AIC. As exemplified by similar approaches in yeast, Drosophila and C. elegans, the efficient zebrafish model brings this powerful forward genetic tool to a vertebrate. Without any *a priori* assumption, novel genes and signaling pathways will be discovered. Based on the pilot screen, whereby 4 AIC modifiers were identified by screening 669 candidate GBT lines (86), it is estimated that ∼200 genetic modifiers of AIC will be resulted, if the screen covers the whole zebrafish genome. Detailed studies of these AIC modifiers would generate a comprehensive genetic landscape of pathophysiology of AIC.
2) The reverse genetic tools in zebrafish will continue to uncover new mechanisms and discover new therapeutic target genes. While some therapeutic target genes were directly suggested by salutary modifiers such as *GBT419/rxraa*, others emerged during detailed mechanistic studies of deleterious modifiers such as *GBT411/DNAJb6 (L)* (86). Gene knockout mutants can be conveniently generated using genome editing technology, including transcription activator-like effector nucleases (TALENs) and clustered regularly interspaced short palindromic repeats/CRISPR associated proteins technologies (CRISPR/Cas9) (97–99). Gain-of-function studies can also be efficiently carried in zebrafish, and spatiotemporal regulation of gene expression can be achieved by adding binary control elements such as GAL4/UAS or Cre/LoxP system (96). More myocardial and/or endocardium-based genes and signaling pathways will be uncovered for AIC, and more therapeutic strategies that target at either the early or late phase of AIC will be discovered, respectively.
3) Blessed with powerful chemical genetics, Zebrafish would accelerate the discovery of effective therapeutic compounds for AIC. A zebrafish-mouse drug screen platform has been proven to be effective in prioritizing therapeutic compounds (66). Because of its small body size, much less compounds are needed when testing in zebrafish than in rodents. Consequently, much higher throughput can be achieved by using this platform. New therapeutic compounds will be benchmarked with existing therapies based on their therapeutic efficacy.

The major limitation for zebrafish as a disease model is associated with its identity as a lower vertebrate model. Some genes and related mechanisms might only occur in the fish model, but not be in humans. Therefore, it would be important to integrate zebrafish with other *in vivo* and *in vitro* experimental models. For example, more detailed mechanistic studies could benefit from additional studies in rodents that have more sophisticated genetic tools, as well as cell culture models for deciphering signaling pathways. New therapeutic strategies discovered from zebrafish need to be validated in larger mammalian models, and finally in AIC patients. Nevertheless, there is compelling reason to anticipate that the next decade will witness even more significant contribution of the zebrafish model to both mechanistic studies and therapeutic development of AIC.
